# Differences in Intertidal Microbial Assemblages on Urban Structures and Natural Rocky Reef

**DOI:** 10.3389/fmicb.2015.01276

**Published:** 2015-11-20

**Authors:** Elisa L.-Y. Tan, Mariana Mayer-Pinto, Emma L. Johnston, Katherine A. Dafforn

**Affiliations:** ^1^Evolution and Ecology Research Centre, School of Biological, Earth and Environmental Sciences, University of New South Wales, SydneyNSW, Australia; ^2^Sydney Institute of Marine Science, MosmanNSW, Australia

**Keywords:** biofilm, artificial structures, rocky shores, seawalls, 16S rRNA sequencing, Sydney Harbour

## Abstract

Global seascapes are increasingly modified to support high levels of human activity in the coastal zone. Modifications include the addition of defense structures and boating infrastructure, such as seawalls and marinas that replace natural habitats. Artificial structures support different macrofaunal communities to those found on natural rocky shores; however, little is known about differences in microbial community structure or function in urban seascapes. Understanding how artificial constructions in marine environments influence microbial communities is important as these assemblages contribute to many basic ecological processes. In this study, the bacterial communities of intertidal biofilms were compared between artificial structures (seawalls) and natural habitats (rocky shores) within Sydney Harbour. Plots were cleared on each type of habitat at eight locations. After 3 weeks the newly formed biofilm was sampled and the 16S rRNA gene sequenced using the Illumina Miseq platform. To account for differences in orientation and substrate material between seawalls and rocky shores that might have influenced our survey, we also deployed recruitment blocks next to the habitats at all locations for 3 weeks and then sampled and sequenced their microbial communities. Intertidal bacterial community structure sampled from plots differed between seawalls and rocky shores, but when substrate material, age and orientation were kept constant (with recruitment blocks) then bacterial communities were similar in composition and structure among habitats. This suggests that changes in bacterial communities on seawalls are not related to environmental differences between locations, but may be related to other intrinsic factors that differ between the habitats such as orientation, complexity, or predation. This is one of the first comparisons of intertidal microbial communities on natural and artificial surfaces and illustrates substantial ecological differences with potential consequences for biofilm function and the recruitment of macrofauna.

## Introduction

Coastal zones have great socioeconomic value supporting industry, trade, and growing urban populations. These systems also support great biological diversity and provide important ecosystem services such as nutrient cycling and food (Costanza et al., 1997; Ortega-Morales et al., 2010). Increasingly, urbanization and industrialization are transforming coastal zones around the globe, and a substantial literature now documents the ecological changes to plant and animal communities associated with this physical transformation (Blockley, 2007; Glasby et al., 2007; Bulleri and Chapman, 2010). However, advances in molecular biology have only recently enabled us to study the impact that intense coastal development is having on the largely hidden, yet ecologically important, microbial communities.

More than 50% of the coastline has been modified in some regions of Japan (Koike, 1996), Australia (Chapman and Bulleri, 2003), USA (Davis et al., 2002), and Europe (Airoldi et al., 2005; Airoldi and Beck, 2007). Modifications include the addition of infrastructure to support activities, such as commercial and recreational boating, fishing, tourism, and waterside living (Airoldi and Bulleri, 2011; e.g., jetties, pilings, and pontoons) and coastal defense structures (e.g., seawalls and breakwaters; Vaselli et al., 2008; Browne and Chapman, 2011). This extensive coastal armouring has significant local and regional effects on natural systems, from loss of natural habitats, decreases in diversity, and increase in non-indigenous species to homogenization of systems (e.g., Dafforn et al., 2013).

Artificial structures usually differ from natural habitats in their substrate material, substrate age, orientation, shading, and habitat complexity (Glasby and Connell, 2001; Bulleri and Chapman, 2004; Bellou et al., 2012). Seawalls, for instance, generally have homogenous surfaces compared to natural rocky shores as they lack microhabitats such as rock pools, crevices, and overhangs (Chapman, 2006). Furthermore, as opposed to the relatively horizontal natural rocky shores, seawalls are often built vertically and have a steep slope (Chapman, 2006). Consequently, although they can support relative diverse assemblages of organisms, they do not mimic natural habitats and there is substantial evidence that they do not function as surrogates for rocky shores (Glasby and Connell, 1999; Bulleri and Chapman, 2010). While we have achieved substantial advances in understanding how and why macrofaunal communities differ between natural habitats and artificial structures (Glasby and Connell, 2001; Blockley, 2007; Glasby et al., 2007), the consequences of this habitat modification for microbial communities remain largely unknown.

The earliest microbial colonizers of any surface form biofilms (Dang and Lovell, 2000; Davey and O’Toole, 2000; Wahl et al., 2012) and their formation can be divided into several stages. Physico-chemical interactions allow for initial cell attachment on surfaces (e.g., plant surfaces, plastic, and sediment particles) and a cell monolayer is formed (Decho, 2000). Cells in the monolayer undergo proliferation, attracting other microbes for attachment, forming an active biofilm of microcolonies. Development of a mature biofilm through secretion of a matrix of mucilaginous extracellular polymers allows for cells to become motile and undergo chemotaxis. As a result, spreading of biomass and horizontal gene transfer occurs (Henschel and Cook, 1990; Singh et al., 2006). These complex aggregates of microbes consisting of mucus, microalgae, and bacteria (Wahl, 1989; Decho, 2000; Ortega-Morales et al., 2010) form the basis of many ecological processes that maintain the biosphere such as biogeochemical cycling of carbon and nutrients (Azam et al., 1993; Narváez-Zapata et al., 2005), primary production in intertidal systems (Moriarty, 1997; Decho, 2000), organic matter degradation (Davey and O’Toole, 2000), contaminant remediation (Singh et al., 2006) and trophic linkage (Ortega-Morales et al., 2010).

In marine systems, biofilms have at least three clearly defined roles – (1) as settlement, attachment and metamorphosis cues for a variety of sessile marine invertebrate larval (Holmström et al., 1992; Wieczorek and Todd, 1997; Thompson et al., 2005), such as mussels (Yang et al., 2014) and bryozoans (Dahms et al., 2004); (2) as primary attachment sites for plant and animal propagules (Wahl, 1989); and (3) the basis of intertidal food webs, as it is the major source of primary production and the largest biomass consumed *in situ*, in particular by grazers (Thompson et al., 2000, 2005). Due to their short generation time, biofilm bacteria are often at the forefront in responding to and recovering from environmental stressors (Lowe and Pan, 1996), altering their community composition and relative abundance in response to changes (Nocker et al., 2004). For example, biofilms are extremely sensitive to environmental changes – such as contamination variations in pH, nutrient and oxygen availability (Singh et al., 2006; Lear and Lewis, 2009; Mayer-Pinto et al., 2011).

Artificial structures may affect microbial communities directly through abiotic factors such as hydrodynamics, changing contaminant concentrations, material, shading, and habitat complexity (Geesey et al., 1992; Stoodley et al., 1998; Davey and O’Toole, 2000). Past studies have shown that biofilms that were exposed to laminar flows developed a different morphology to those exposed to turbulent flows. An increase in biofilm biomass has also been linked to increase in nutrient (carbon and nitrogen) and metal (zinc) concentrations (Stoodley et al., 1998; Mayer-Pinto et al., 2011). Artificial structures may also affect microbial communities indirectly via the assemblages that such structures support, e.g., grazers (Davey and O’Toole, 2000; Skov et al., 2010). Although there is direct removal of biofilm by grazing, there is also the potential for grazing to boost the photo-autotrophic biomass of biofilms by removing the biofilm canopy and allowing greater light and nutrient penetration (Skov et al., 2010).

Studies of microbial community response to anthropogenic environmental change have given particular focus to artificial substrates in freshwater systems (Tien et al., 2009) and estuaries (Jones et al., 2007); while studies of artificial structures have identified changes to intertidal macrofauna compared with natural habitats (Bulleri and Chapman, 2004; Chapman, 2006; Bilkovic and Mitchell, 2013). Microbial biofilm formation and factors that regulate their assemblages have also been conducted on rocky shores (Thompson et al., 2004, 2005; Narváez-Zapata et al., 2005). However, no previous study has used amplicon sequencing to compare bacterial communities between artificial structures and natural shores and investigate the effects of increasing marine urbanization. Amplicon sequencing is a powerful tool for assessing structural changes in the biofilm communities colonizing artificial structures and the potential functional consequences (DeLong et al., 2006). We investigated whether potential structural changes to biofilm communities are related to the habitats or the local environmental conditions associated with the habitats. Microbial communities were identified using amplicon sequencing of the 16S rRNA gene to target bacteria, which form the primary component of biofilms (Davey and O’Toole, 2000).

## Materials and Methods

### Experimental Design

**FIGURE 1:**
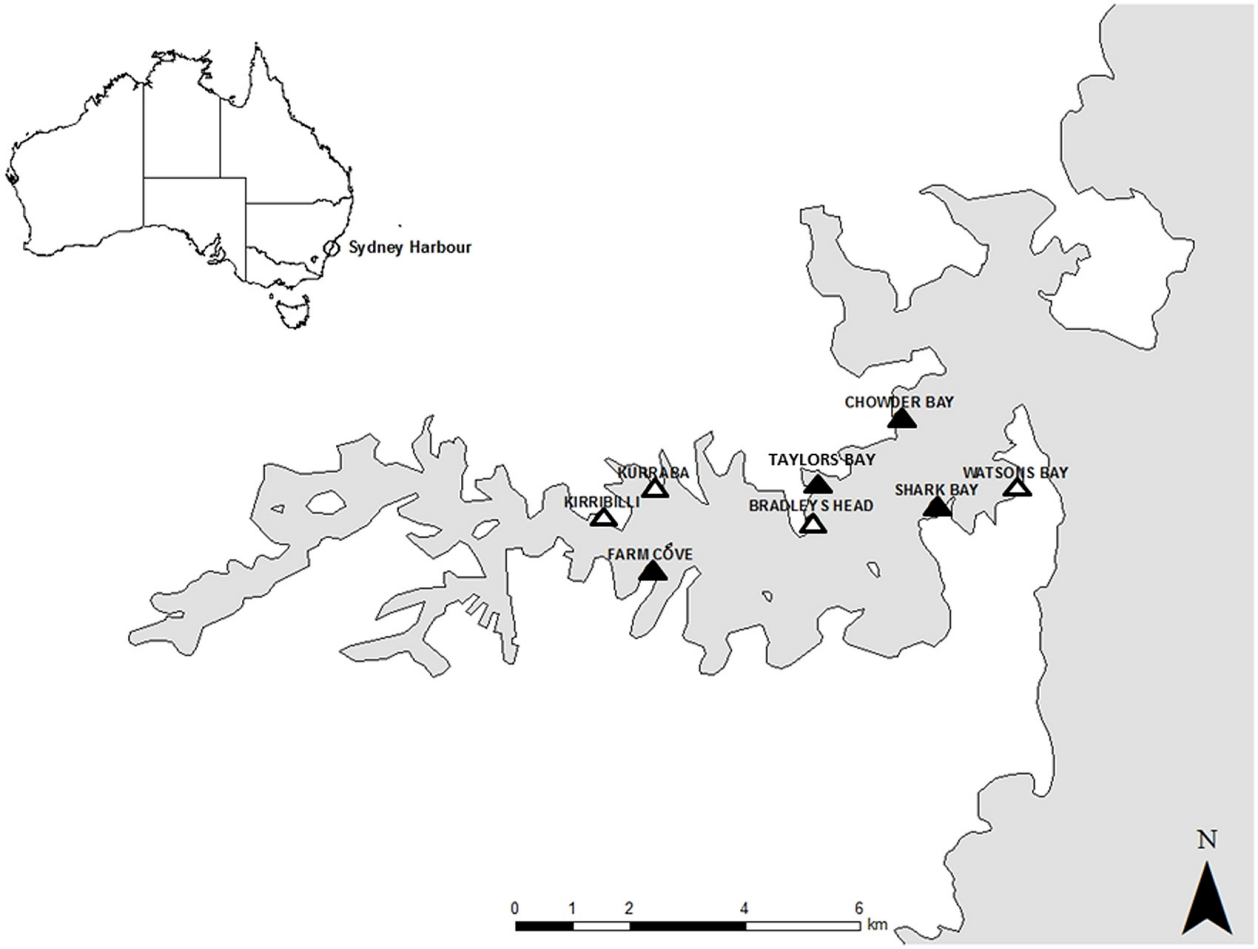
**Map of Sydney Harbour with the studied locations.** Natural rocky shores were studied at Taylors Bay, Chowder Bay, Farm Cove, and Shark Bay. Seawalls were studied at Bradleys Head, Kirribilli, Kurraba, and Watsons Bay.

The study was conducted in Sydney Harbour, New South Wales, Australia in the austral winter of 2014. Sydney Harbour is a highly urbanized area, with approximately half of the harbour’s foreshore replaced by seawalls (Blockley, 2007). Sampling was done in the intertidal zone from two types of habitat; natural rocky shores and artificial seawalls. Four locations of each type of habitat were selected for this study based on their availability within the harbour and substantial effort was made to ensure that seawalls and rocky shores were interspersed. Natural rocky shores were studied at Taylors Bay, Chowder Bay, Farm Cove, and Shark Bay while studies on artificial seawalls were located at Bradleys Head, Kirribilli, Kurraba, and Watsons Bay (**Figure 1**). Seawalls were all vertical while rocky shores were mostly horizontal with a gentle slope (<20°) because this is the most common type of rocky shore in Sydney Harbour.

This study investigated the variation in bacterial communities on the surface of natural rocky shores and artificial seawalls using recently cleared plots of existing surfaces and deployed surfaces (recruitment blocks). Six 10 cm × 10 cm plots were randomly chosen and cleared at each type of habitat, in each location, close to the high water mark, 0.8–1.1 m from the low tide line. Plots were marked using 6.5 mm raw plugs drilled at two opposing corners of each plot. All organisms found within the plot were removed using a hammer and a chisel. Plots were cleared thoroughly by scraping and scrubbing with an iron brush to ensure removal of all organisms and the existing biofilm on the substrata. This was done to ensure consistency of biofilm age across all samples. Newly formed biofilms were sampled 3 weeks after clearing. Mature biofilms are formed within days (Singh et al., 2006) and pilot setup of the experiment was tested to determine a suitable time frame for this study. At the same time that plots were cleared, four “recruitment blocks” were deployed for 3 weeks at each location to standardize the time frame available for biofilm colonization on all surfaces. Each “recruitment block” consisted of two 390 long × 140 deep × 190 mm high concrete bricks that were fastened together using cable ties to increase weighting and reduce the chance that replicates would be lost in high wave action. Blocks were placed adjacent to artificial or natural habitats and within 5 m of the nearest scraped plot. Recruitment blocks were deployed to account for possible differences in communities associated with habitat orientation, substrate material and age, as well as the local history of the natural substrata found at each location.

### Environmental Parameters

Environmental parameters including temperature, salinity, dissolved oxygen (DO), and pH were measured adjacent to seawalls and rocky shores during the high tide immediately after biofilm samples were collected from the plots to investigate abiotic differences that might influence bacterial communities. Three replicate measurements were collected *in situ* at all locations using a water quality probe calibrated prior to data collection (YSI-Sonde 6600-v2, Yellow Springs, OH, USA).

### Bacterial Biofilm Community

Three weeks after set-up and deployment, a randomly selected 3 cm × 3 cm area (Thompson et al., 2004, 2005) of biofilm was swabbed for 20 s from the upper horizontal surface of each recruitment block and cleared plot at each location using sterile cotton tips. All biofilm samples were collected within 1–2 h of low tide to ensure evenness of residual seawater across all samples. Swabs from the cleared plots were randomly pooled into three replicates (each replicate consists of swabs from two separate plots). This was done because single replicate plots yielded insufficient DNA for individual sequencing. Swabs from recruitment blocks yielded sufficient DNA material for sequencing and were therefore not pooled. This provided four replicates from each location. Swabs were immediately placed in separate cryogenic vials and stored in liquid nitrogen in the field then frozen at -80°C until DNA extraction, which was done within 2 weeks of sampling. Genomic DNA was extracted with the PowerBiofilm^®^ DNA Isolation Kit (Mo Bio Laboratories Inc., Carlsbad, CA, USA), according to the manufacturer’s instructions.

Amplicon sequencing of the 16S rRNA gene was done at the Molecular Research DNA Lab (MR DNA; Shallowater, TX, USA). Bacterial 16S primers 104F (Bertilsson et al., 2002) and 530R (Lane, 1991) were used to generate amplicon libraries for paired-end sequencing on the Illumina Miseq platform. Analysis of paired-end sequence data was processed using a proprietary analysis pipeline (MR DNA, Shallowater, TX, USA). Briefly, barcodes attached to sequences were removed. Sequence data underwent denoising and sequences shorter than 150 base pairs in length, and those with ambiguous bases and homopolymers exceeding 8 were removed to generate operational taxonomic units (OTUs). Next, chimeric sequences were identified and removed. OTUs were clustered at 3% divergence (97% similarity) to remove potential errors in sequence data. Taxonomy for the remaining OTUs was classified using the sequence alignment tool BLASTn against a curated GreenGenes database (DeSantis et al., 2006). Chloroplasts and OTUs with ≤5 occurrences were removed. This provided a total of 6497 OTUs sampled from plots and 8937 OTUs sampled from recruitment blocks.

### Statistical Analyses

The factors considered in the analyses were Habitat (fixed, two levels – natural rocky shores or seawalls) and Locations (random, four levels and nested within habitat). Due to differences in sampling methodology, bacterial community data collected were analyzed separately for plots and recruitment blocks and used to construct resemblance matrices using the Bray–Curtis dissimilarity index for a relative abundance-weighted measure of how similar the bacterial communities are in terms of their community structure (i.e., relative abundance and composition of species). Resemblance matrices were also constructed using Jaccard similarity index, for a comparison between communities based solely on the presence and absence of bacterial OTUs, for a measure of bacterial community composition. Principal co-ordinates analysis (PCO) was done to visualize the multivariate patterns in biofilm bacterial community structure and composition based on the data generated from each biofilm sample. Vector plots were overlaid to illustrate the relationship (*R* > 0.5) of bacterial classes to differences. Differences in bacterial community structure and composition were investigated with permutational multivariate analysis of variance (perMANOVA). Where significant differences were observed among habitats, the contribution of each bacterial class to the similarity/dissimilarity within/between habitats was further investigated with similarity percentage analysis (SIMPER; Clarke and Warwick, 2001; Clarke and Gorley, 2006).

Data were also rarefied to a common number of OTUs to investigate differences in diversity among habitats. Briefly, the lowest number of OTUs found in any one sample was assessed (770 OTUs) and the datasets were randomly subsampled to a common number of OTUs for a comparison of both alpha (OTU (species) richness, Shannon’s diversity and Pielou’s evenness) and beta (dispersion) diversity among habitats. Univariate diversity data, environmental parameters, were analyzed with permutational analysis of variance (perANOVA), using Euclidean distance. Beta diversity was calculated as the mean distance of individual observations for habitat to the group centroid for both community structure (Bray–Curtis) and community composition (Jaccard) using permutational analysis of multivariate dispersions (PERMDISP; Anderson, 2006).

All perMANOVAs were performed using Type III sum of squares to account for unbalanced data and 9999 permutations of residuals under a reduced model for raw data. Heterogeneity of dispersions was tested using PERMDisp. All data were analyzed using PRIMER-6 software (Clarke and Gorley, 2006) and its PERMANOVA+ add-on (Anderson et al., 2008).

## Results

### Environmental Parameters

Temperature (°C; **Supplementary Figure S1A**), salinity (psu; **Supplementary Figure S1B**), DO (mg/L; **Supplementary Figure S1C**) and pH (**Supplementary Figure S1D**) measurements did not range widely and did not differ significantly between seawalls and rocky shores, but were variable among locations (**Table 1**). Temperature ranged from 17.6 to 18.8°C, while salinity ranged from 35.0 to 36.0 psu. DO varied from 12.2 to 15.1 mg/L and pH values varied from 8.3 to 9.2.

**Table 1:** Permutational multivariate analysis of variance (perMANOVA) comparing environmental parameters (A) Temperature, (B) Salinity, (C) Dissolved Oxygen, (D) pH sampled at high tide from natural rocky shores and artificial seawalls.

Source	df	MS	*Pseudo-F*		MS	*Pseudo-F*	
		**(A) Temperature**			**(B) Salinity**		
			
Ha	1	0.13	0.03	ns	6.74	2.86	ns
Lo (Ha)	6	3.56	37.54	^∗∗^	2.36	17.74	^∗∗^
Residual	16	0.09			0.13		
		
		**(C) Dissolved oxygen**			**(D) pH**		
			
Ha	1	0.25	0.08	ns	0.77	0.24	ns
Lo (Ha)	6	3.07	11.32	^∗∗^	3.19	16.58	^∗∗^
Residual	16	0.27			0.19		


*Factors include Habitat (Ha: fixed, 2 levels) and Locations (Lo: random, 4 levels, nested in habitat). Ns = not significant at *p* < 0.05; ^∗∗^*p* < 0.01.*

### Bacterial Biofilm Community

**FIGURE 2:**
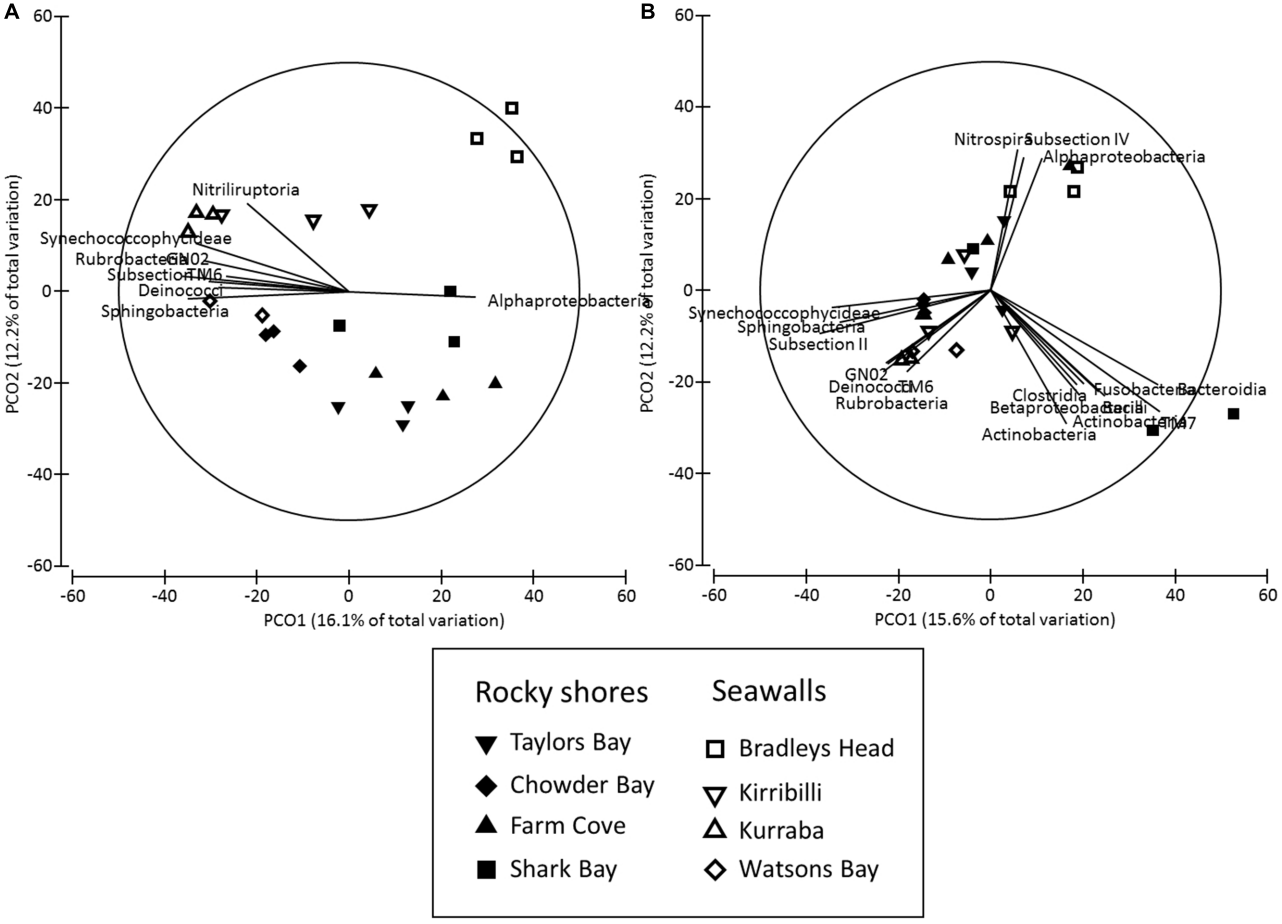
**Principal co-ordinates (PCO) analysis of bacterial community structure (**(A)** Bray – Curtis – relative abundance and composition of species) and composition (**(B)** Jaccard – presence absence data only) sampled directly from natural rocky shores and artificial seawalls (Plots).** Vector plot of bacterial taxa (at level of class) most strongly related (*R* > 0.5) to differences in community structures are also presented. Lengths of vectors indicate the strength and direction of relationships to measured variables.

The structure of microbial communities (i.e., relative abundance and composition of OTUs) on seawalls was different (SIMPER: 84.89%) to those sampled on rocky shores (Ha: *Pseudo-F* = 1.35, *P* < 0.05 **Table 2**, **Figure 2A**) and varied significantly among locations within habitat types [Lo (Ha): *Pseudo-F* = 3.28, *P* < 0.01; **Table 2**, **Figure 2A**]. Communities within rocky shore plots were less similar (SIMPER: 19.63%) than communities within seawalls (24.25%). Differences in structure among habitats were separated along the PCO2 axis with seawalls positively correlated to PCO2 and rocky shores negatively correlated to PCO2 (**Figure 2A**). When the vector plot was overlaid the bacterial classes contributing most to structural differences separated out along the PCO1 axis. This suggests that the bacterial classes identified by the vector analysis primarily explained variation among locations that also separated out along PCO1, with the bacterial class *Alphaproteobacteria* most abundant in locations including Bradleys Head, Shark Bay, and Farm Cove (**Figure 2A**). The bacterial classes *Nitriliruptoria, Synechococcophycideae, Rubrobacteria, GN02, Subsection II, Deinococci*, and *Sphingobacteria* were negatively correlated with PCO1 axis indicating an increased abundance in Kurraba and Watsons Bay (**Figure 2A**).

**Table 2:** perMANOVA comparing bacterial community structure (Bray–Curtis) and composition (Jaccard) sampled directly from natural rocky shores and artificial seawalls ((A) Plots) and from substrate experimentally deployed in the habitat ((B) Recruitment blocks).

		Bray–Curtis			Jaccard		
			
Source	df	MS	*Psuedo-F*			MS	*Pseudo-F*	
**(A) Plots**
Ha	1	8434	1.35	^∗^		3769	1.25	ns	
Lo (Ha)	6	6296	3.28	^∗∗^		3035	1.89	^∗∗^	
Residual	15	1917				1607			
**(B) Recruitment blocks**
Ha	1	6834	1.16	ns		3666	0.92	ns	
Lo (Ha)	6	35228	2.23	^∗∗^		23898	2.01	^∗∗^	
Residual	24	63118				47548			


*Factors include Habitat (Ha: fixed, 2 levels) and Locations (Lo: random, 4 levels, nested in habitat).*

*Ns = not significant at *p* < 0.05; ^∗^*p* < 0.05; ^∗∗^*p* < 0.01.*

SIMPER analysis indicated that 10 bacterial classes contributed to 92.52% of the dissimilarity among habitats sampled from plots (**Table 3**). Most of the dissimilarity was explained by the *Alphaproteobacteria* (28.31%)*, Subsection II* (20.13%), and *Bacilli* (10.02%) classes (**Table 3**). *Alphaproteobacteria, Bacilli, Gammaproteobacteria, Oscillatoriophycideae, Subsection IV, Flavobacteria*, and *Deltaproteobacteria* were most abundant on rocky shores, while *Subsection II, Synechococcophycideae*, and *Sphingobacteria* were most abundant on seawalls (**Table 3**). These taxa contributed to 19.43% of the dissimilarity among habitats. Bacterial community composition (based on presence/absence data only) differed only among locations and was not significantly different between plots sampled from seawalls and from rocky shores [Ha: *Pseudo-F* = 1.25, *P* > 0.05; Lo (Ha): *Pseudo-F* = 1.89, *P* < 0.01; **Table 2**, **Figure 2B**].

**Table 3:** Results of SIMPER analysis giving dissimilarities among habitats sampled from plots.

Plots	Rocky shores	Seawalls				

**Class**	**Av. Abund**	**Av. Abund**	**Av.Diss**	**Diss/*SD***	**Contrib%**	**Cum.%**
Alphaproteobacteria	**29359.08**	28033.45	12.14	1.29	28.31	28.31
Subsection II	17065.17	**20611.73**	8.63	1.3	20.13	48.44
Bacilli	**7725.08**	131.91	4.3	0.33	10.02	58.45
Gammaproteobacteria	**10450.33**	9746.82	3.46	1.13	8.07	66.53
Oscillatoriophycideae	**4432.08**	4263.45	2.77	1.16	6.46	72.99
Subsection IV	**2754**	1824.55	1.86	0.84	4.33	77.31
Synechococcophycideae	2942.42	**4126.82**	1.79	1.32	4.17	81.48
Flavobacteria	**4506.42**	3797.64	1.62	1.14	3.77	85.25
Sphingobacteria	2764.33	**3794.09**	1.57	1.3	3.66	88.91
Deltaproteobacteria	**3385.92**	1647.45	1.55	1.02	3.61	92.52


*Listed are the 10 bacterial classes contributing most to the dissimilarity with respect to average abundance (Av. Abund), average dissimilarity (Av. Diss), quotient of dissimilarity and standard deviation (Diss/SD), % contribution to differences (Contrib%) and cumulative % contribution to differences (Cum.%). Abundance values highlighted in bold indicate the highest abundance of that class in a particular habitat.*

**FIGURE 3:**
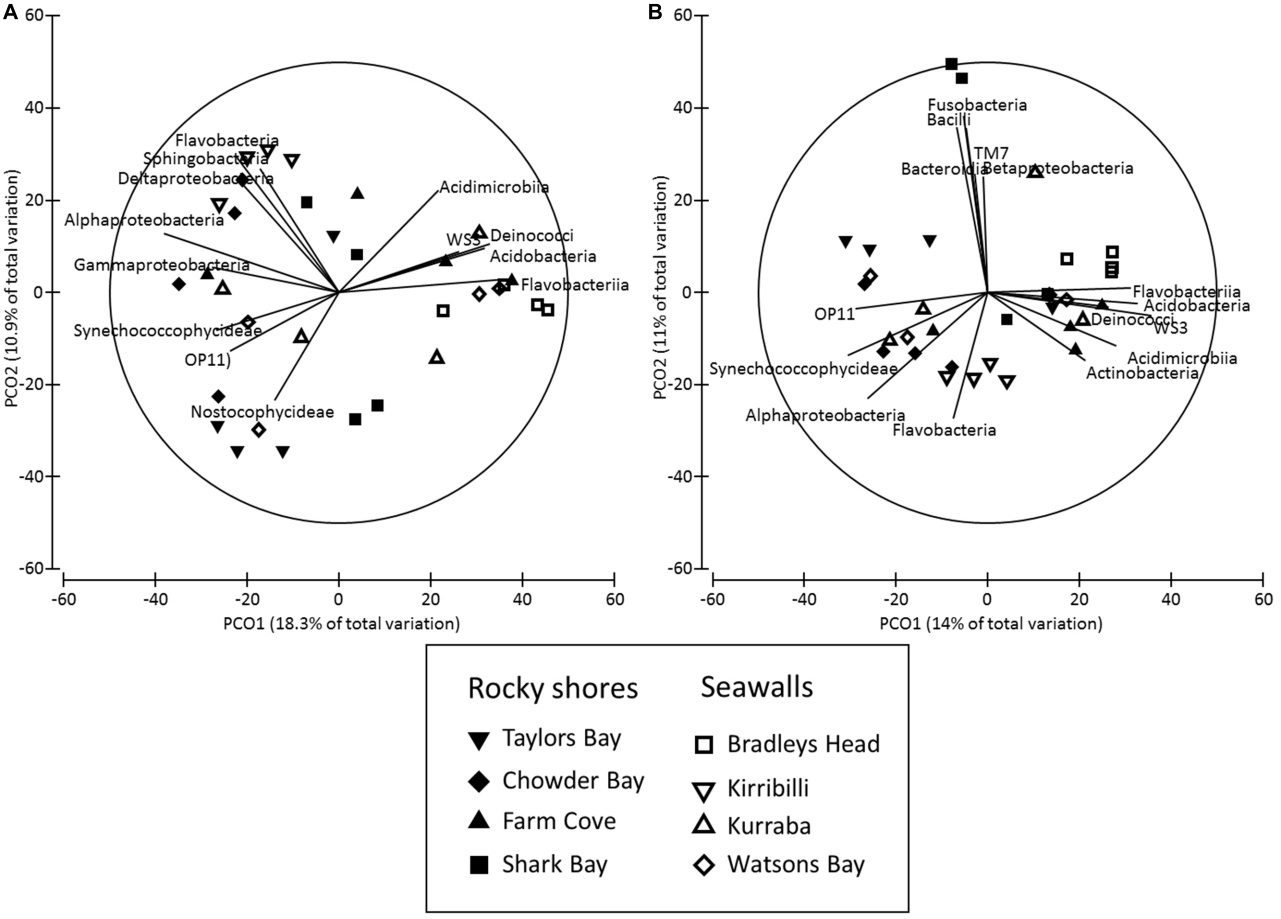
**Principal co-ordinates (PCO) analysis of bacterial community structure (**(A)** Bray–Curtis) and composition (**(B)** Jaccard) sampled from substrate experimentally deployed in natural rocky shores and artificial seawalls (Recruitment blocks).** Vector plot of bacterial taxa (at level of class) most strongly related (*R* > 0.5) to differences in community structures are also presented. Lengths of vectors indicate the strength and direction of relationships to measured variables.

Both the structure and composition of bacterial communities recruiting to deployed blocks did not differ significantly between seawalls and rocky shores (*P* > 0.05, **Table 2**, **Figures 3A,B**), only among locations (*P* < 0.01, **Table 2**).

**FIGURE 4:**
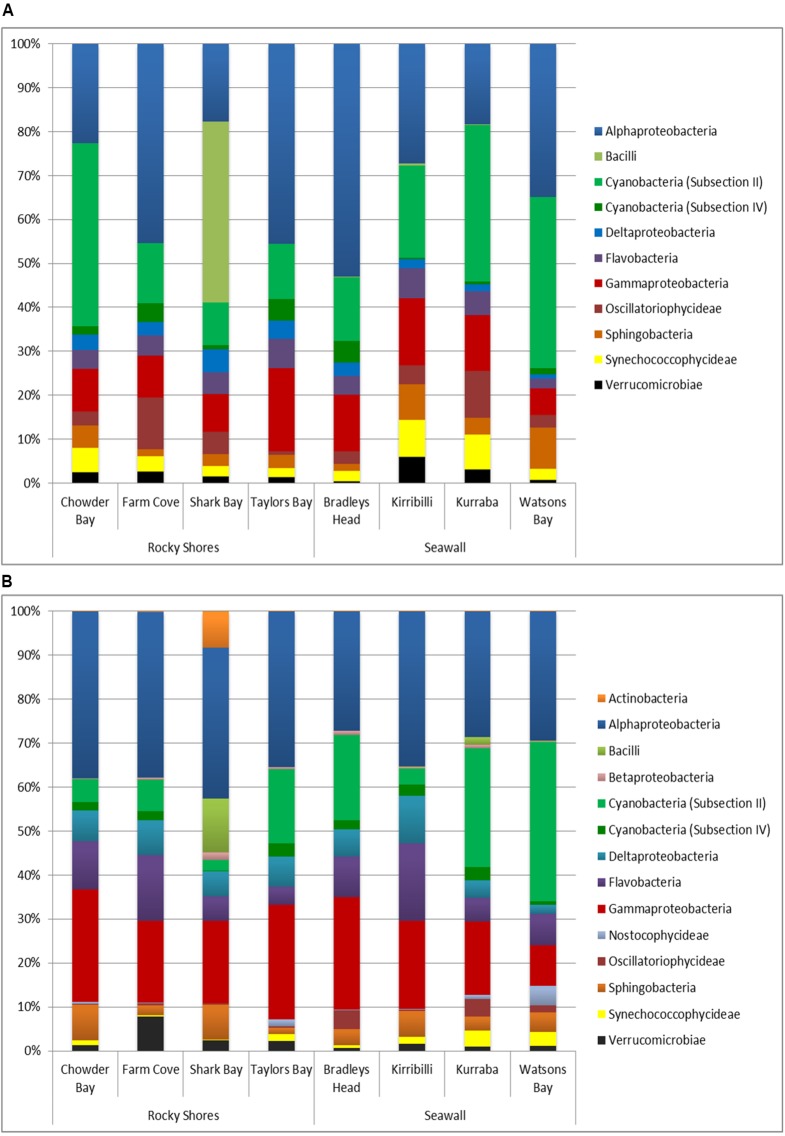
**Bacterial dominance grouped by Class sampled directly from natural rocky shores and artificial seawalls ((A) Plots) and from substrate experimentally deployed in the habitat ((B) Recruitment blocks)**.

Overall, biofilm communities sampled from both plots and blocks were dominated by *Alphaproteobacteria* (33.3 and 33.1% respectively, **Figures 4A,B**). *Gammaproteobacteria* (11.5% of plots, 19.8% of blocks) and *Cyanobacteria (Subsection II;* 22.4% of plots, 12.9% of blocks) were also major components of the biofilm community sampled from seawalls and rocky shores (**Figures 4A,B**).

**FIGURE 5:**
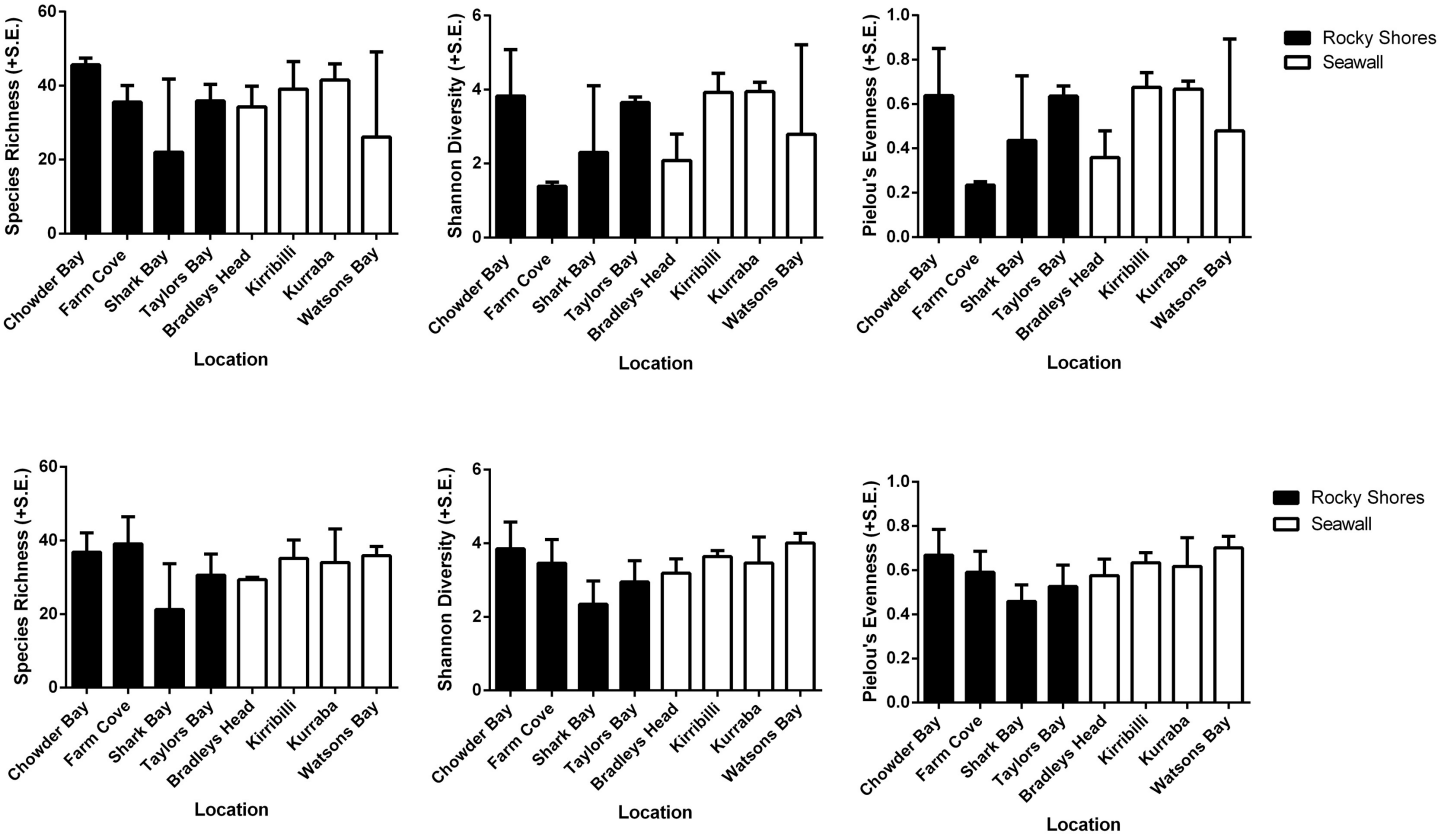
**Species richness, Shannon’s diversity and Pielou’s evenness analyzed from rarefied datasets of plots **(Top)** and recruitment blocks (Bottom)**.

Alpha diversity (species richness, Shannon’s diversity, and Pielou’s evenness) did not differ among habitats for both plot and recruitment blocks (*P* > 0.05, **Table 4**, **Figure 5**). Beta diversity for both community structure (PERMDisp *P* = 0.2292 > 0.05) and community composition (PERMDisp *P* = 0.3494 > 0.05) on plots also did not differ between seawalls and rocky shores. Similarly, beta diversity on recruitment blocks for both community structure (PERMDisp *P* = 0.5706 > 0.05) and composition (PERMDisp *P* = 0.2482 > 0.05) did not differ between habitats.

**Table 4:** perMANOVA comparing alpha diversity (species richness, Shannon’s diversity, Pielou’s evenness) sampled directly from natural rocky shores and artificial seawalls ((A) Plots) and from substrate experimentally deployed in the habitat ((B) Recruitment blocks).

		Species richness			Shannon diversity			Pielou evenness		
				
Source	df	MS	*Pseudo-F*		MS	*Pseudo-F*		MS	*Pseudo-F*	
**(A) Plots**						
Ha	1	80.14	0.52	ns	3.13	0.94	ns	8.01E-2	0.86	ns
Lo(Ha)	6	154.94	2.10	ns	3.36	4.40	^∗^	9.44E-2	4.62	^∗^
Residual	15	73.89			0.76			2.04E-2		
**(B) Recruitment blocks**						
Ha	1	68.22	0.42	ns	2.04	2.03	ns	5.33E-2	2.73	ns
Lo(Ha)	6	161.72	3.86	^∗∗^	1.01	3.38	^∗^	1.95E-2	2.43	ns
Residual	24	41.87			0.30			8.03E-3		


*Factors include Habitat (Ha: fixed, 2 levels) and Locations (Lo: random, 4 levels, nested in habitat). Ns = not significant at *p* < 0.05; ^∗^*p* < 0.05; ^∗∗^*p* < 0.01.*

Seven bacterial phyla recruited to the deployed blocks that were not present in plots sampled directly from seawalls and rocky shores (**Supplementary Table S1**). This included the phyla *Aquificae, Chlorobi, KSB1, NC10, NKB10, SBR1093*, and *WS3* (**Supplementary Table S1**).

## Discussion

Our results showed that natural rocky shores and seawalls were colonized by similar bacterial taxa, but in different abundances. Much of the structural difference could be explained by greater relative abundances of *Proteobacteria* on rocky shores than seawalls. We also found that bacterial community composition and structure did not differ between artificial and natural habitats when differences in substrate orientation and surface were removed through the deployment of standard recruitment blocks. In addition, temperature, salinity, DO, and pH did not differ between types of habitats. Differences found in the intertidal bacterial communities are, therefore, likely due to intrinsic differences between artificial and natural habitats, e.g., surface complexity [hardness and texture (Coombes et al., 2011)] or biotic factors, rather than differences in the local environment, such as wave energy, water quality or other physico-chemical variables. Indeed, temperature, salinity, DO, and pH did not differ between types of habitats, so differences cannot be attributed to these environmental factors. The current study, however, did not quantify hydrodynamics and nutrient concentrations; thus there is a possibility that local flow regimes and nutrient concentrations were contributing drivers of the differences found in community structure.

### Bacterial Community Differences between Natural and Artificial Habitats

Microbial biofilms play an important role in biogeochemical processes. Differential abundances of bacterial taxa on natural rocky shores and artificial seawalls can have potential changes to function and influence on overall biological processes. Heterotrophic bacteria such as members of the *Proteobacteria* (e.g., *Alpha-, Gamma-*, and *Delta-proteobacteria*) are metabolically extremely diverse (Tujula et al., 2010). They are crucial to nutrient cycling and perform transformations and remineralization of material such as organic carbon and nitrogen (Azam et al., 1993; Azam, 1998). In the current study, all of these classes of *Proteobacteria* were relatively more abundant on natural rocky shores than seawalls. *Alphaproteobacteria* dominated the biofilm communities in this study and are often highlighted as the primary colonizing group of hard substrates, but are relatively less abundant in soft sediments. Given that a major impact of increasing marine urbanization is the replacement of soft sediments with artificial hard structures (Airoldi et al., 2009; Dafforn et al., 2013), this has the potential to alter the overall abundance of *Alphaproteobacteria* in the ecosystem. However, as highlighted in this study, seawalls may not provide a surrogate for rocky shores with respect to the bacterial taxa they support. While *Alphaproteobacteria* dominated the seawall biofilms, they were still relatively less abundant than on rocky shores. This may represent reduced biogeochemical processing of carbon and nitrogen although further investigation, e.g., using metagenomics tools (Dinsdale et al., 2008) would be required to determine this.

Primary productivity is a principal process occurring in the oceans and represents the first stage in the flow of energy and matter through ocean systems (Cotner and Biddanda, 2002). In most aquatic habitats, the cyanobacteria are responsible for the majority of bacterial primary production (Capone et al., 1997; Liu et al., 1997). In the current study, several classes of *Cyanobacteria* (*Subsection II, Synechococcophycideae, Oscillatoriophycideae, Subsection IV*) were important in differentiating between rocky shore and seawall communities. *Subsection II* (*Pleurocapsales*) cyanobacteria are known to survive extreme desiccation and UV radiation (Billi et al., 2000) and many have evolved salt tolerance mechanisms or actually require salt for growth (Cumbers and Rothschild, 2014). Together with *Synechococcophycideae*, these autotrophic cyanobacteria were more abundant on seawalls. In contrast, *Subsection IV* (*Nostocales*) and *Oscillatoriophycideae* were relatively more abundant on natural rocky shores than artificial seawalls. Members of the *Nostocales* group of cyanobacteria are filamentous and vegetative cells may differentiate into heterocysts that are then important in nitrogen fixation under aerobic conditions (Tomitani et al., 2006). The lower relative abundance of *Subsection IV* on seawalls compared to rocky shores may have implications for nutrient cycling if this translates to reduced potential for nitrogen fixation on artificial structures. However, additional manipulations and measurements would be required to quantify any functional differences. In addition, it has been shown that vegetative cells of the *Subsection IV* cyanobacteria may differentiate into akinetes (resting cells resistant to environmental stress) in response to local conditions (Tomitani et al., 2006). Since intertidal systems represent extreme environments where high temperature, desiccation, high levels of UV-radiation and increased wave action place significant stress on local communities, it remains uncertain which cell type dominates in the *Nostocales* from these assemblages, and how this might translate into potential functions. Having taxa capable of coping with these conditions is important for community resilience. This resilience might be decreased on seawalls, where this taxa was found in lower abundances.

### Ecological Effects of Seawalls

Artificial structures result in the introduction of substrates that are often alien to natural conditions or that differ, for example, with respect to composition, age, orientation, and material (Glasby, 1999c, 2000, 2001). The uniformity of construction has been shown to result in homogeneity across terrestrial landscapes (McKinney, 2006), although less is known in marine systems. We found little evidence that seawalls supported more homogeneous communities than natural rocky shores. Measures of alpha and beta diversity were similar among habitats, although there was significant variation in species richness, diversity, and evenness among locations. This possibility of biotic homogenisation in the marine environment should be further investigated at higher trophic levels by examining patterns in the macroinvertebrates recruiting to seawalls and other urban structures. The evaluation of macroinvertebrate homogenization is particularly relevant to biofilm homogeneity due to invertebrate grazers that regulates biofilm structure and function as would be discussed below.

Due to the size of many artificial structures, their construction design and orientation, they often have different illumination levels from shading (Glasby, 1999b) and UV exposure. Artificial structures typically have vertical surfaces or horizontal surfaces facing downward and few have a surface that is analogous to horizontal rocky reef. Differences in light between rocky shores and seawalls have been observed in previous studies (Blockley, 2007) with shading by vertical seawalls found to reduce light levels at certain times of the day. Given the abundance of autotrophic bacteria identified in this study, shading by seawalls and the associated reduction in light availability is more likely to have affected biofilms than physico-chemical measures such as temperature and salinity that did not differ among habitats.

The occurrence of several groups of extremophiles in the current study may be linked to the stressful environments represented by intertidal hard substrates (Thompson et al., 2002). The bacterial group *Bacilli* has several representatives that require high temperatures for growth or can survive a range of high and low temperatures (Rothschild and Mancinelli, 2001). In the current study we found *Bacilli* to be relatively more abundant on rocky shores than seawalls and this may reflect increased tolerance to high temperatures and UV radiation on horizontal shores (Thompson et al., 2002). However, the slope of substrates may mitigate these stressors and also affect the relative abundance of bacteria comprising biofilms. Tidal submersion is longer on gently sloping rocky shores than vertical seawalls and may help reduce exposure to stressful environmental conditions (Bulleri and Chapman, 2010) that lead to desiccation. Differences between patterns observed in the microbial community structure may have been stronger had the full range of photosynthetic organisms including diatoms and microalgae been investigated with the bacteria (Consalvey et al., 2005).

Artificial structures such as seawalls constructed in marine environments create islands of hard-substrate (Airoldi and Beck, 2007) and these structures can change local hydrodynamic conditions which may alter the rate at which nutrients and organic material are delivered or entrained (Glasby, 1999a). While these environmental factors were not measured in the current study, the dominance of the *Proteobacteria* groups in all assemblages suggests that nutrient availability could be an important factor influencing the differences in relative abundances of biofilm taxa. Thus, the presence of *Proteobacteria* groups in lower abundances on seawalls suggests that nutrient cycling may be altered. Increasingly, studies seek to measure rates of biogeochemical processes in relation to artificial structures. Indirect effects on nitrogen gas production derived from the facilitation of invasive macroalgae by coastal defense structures have been found (Geraldi et al., 2014). Future studies might consider quantifying the potential for urban structures to support biofilm communities that perform important ecosystem functions.

Artificial structures may affect microbial communities indirectly via the benthic assemblages that such structures support, composed of algae, invertebrates, and fish. Past research has also shown that several species of mobile grazers commonly found on rocky shores are rare or absent from seawalls (Chapman, 2006). Differences in grazer assemblages could have an effect on the relative abundance of bacterial taxa on natural and artificial structures observed in the current study (Skov et al., 2010). Grazing allows direct removal of biofilm as well as the potential bloom in photo-autotrophic biomass when greater light and nutrient penetration are enabled through the removal of biofilm canopy (Skov et al., 2010). Consequently, grazing is an important driver in the structure and function of biofilms (Burns and Ryder, 2001; Thompson et al., 2004) and *Cyanobacteria* have previously been found to be exploited by populations of macrograzers such as echinoderms, polyplacophorans and gastropods (Hutchinson et al., 2006; Liess and Kahlert, 2009). Therefore the differences in relative abundances of *Cyanobacteria* may be related to differential grazing pressure between seawalls and rocky shores. Grazers were not quantified in the current study, but would provide useful information about changes in trophic dynamics related to marine urban seascapes.

## Conclusion

This study found that while the identity of communities in rocky shore and seawall habitats are similar, relative abundances of bacterial taxa differed. These differences were not due to slope, ecological history or material of the structure under study, but are probably a consequence of surface complexity, benthic assemblages and/or environmental variables. As the requirement for coastal defense structures is expected to increase in the coming years with increasing urbanization of the coastal zone, this study forms a crucial baseline of the ecological consequences of urban seascapes at the microbial scale. Thus, investigation of microbial communities on other widespread artificial structures such as pilings and pontoons should also be conducted. Future research of microbial communities in urban seascapes might consider an approach that includes targeted sequencing to investigate the eukaryotic component of the biofilm. Metatranscriptomics or measurements of processes such as productivity, respiration, and nitrogen cycling could also be used to assess functional changes in bacterial communities in response to marine urbanization. Additionally, it would be worthwhile to study the differences in microbial communities between natural and artificial substrates alongside factors that regulate microbial community assemblages such as grazing, flow movements, and light.

## Conflict of Interest Statement

The authors declare that the research was conducted in the absence of any commercial or financial relationships that could be construed as a potential conflict of interest.
