# Supplementary material for: Differences in Intertidal Microbial Assemblages on Urban Structures and Natural Rocky Reef
Source: Front Microbiol. 2015 Nov 20;6:1276. doi: 10.3389/fmicb.2015.01276 (PMC4653414; doi:10.3389/fmicb.2015.01276)
Supplement: Supplementary file 1 [file Data_Sheet_1.DOCX]

Supplementary Material

Differences in intertidal microbial assemblages on urban structures and natural rocky reef

**Elisa L-Y Tan^*^, Mariana Mayer-Pinto, Emma L. Johnston and Katherine A. Dafforn**

*** Correspondence:** Corresponding Author: elisa.tly@hotmail.com

# Supplementary Figures and Tables

## Supplementary Figures


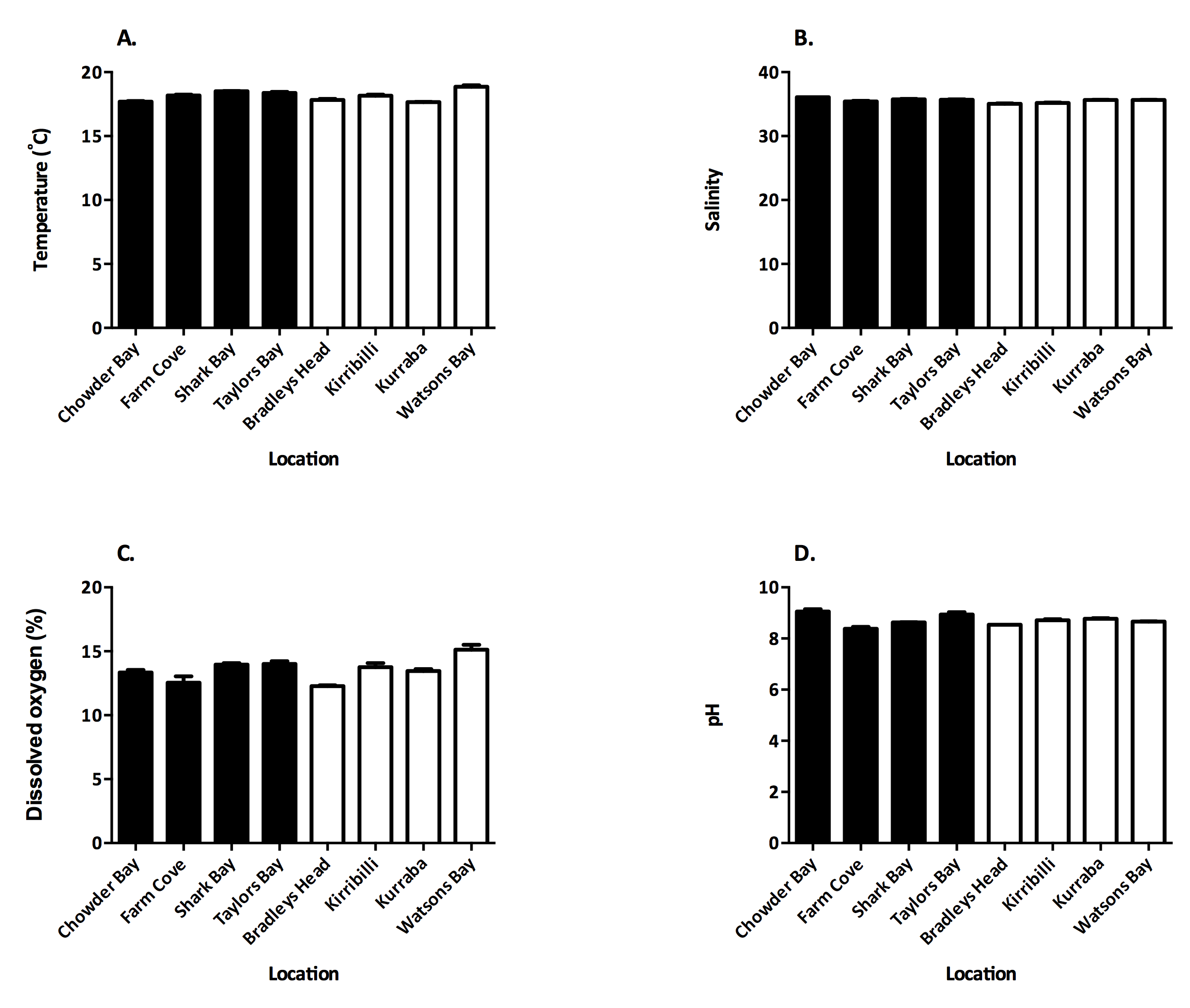


**Supplementary Figure 1.** Environmental parameters (A. Temperature, B. Salinity, C. Dissolved Oxygen, D. pH) +/- S.E. sampled at high tide from natural rocky shores and artificial seawalls. Rocky shores = filled bars and Seawalls = open bars.

## Supplementary Tables

**Supplementary Table 1.** Comparison of the bacterial phyla sampled directly from natural rocky shores and artificial seawalls (A. Plots) and from substrate experimentally deployed in the habitat (B. Recruitment blocks). X = phylum was present.

| **Phyla** | **A. Plots** | **B. Recruitment blocks** |
| --- | --- | --- |
| Acidobacteria | X | X |
| Actinobacteria | X | X |
| Aquificae |  | X |
| Bacteroidetes | X | X |
| Chlorobi |  | X |
| Chloroflexi | X | X |
| Cyanobacteria | X | X |
| Deinococcus Thermus | X | X |
| Fibrobacteres | X | X |
| Firmicutes | X | X |
| Fusobacteria | X | X |
| GN02 | X | X |
| KSB1 |  | X |
| Lentisphaerae | X | X |
| NC10 |  | X |
| Nitrospirae | X | X |
| NKB10 |  | X |
| OP11 | X | X |
| OP3 | X | X |
| Planctomycetes | X | X |
| Proteobacteria | X | X |
| SBR1093 |  | X |
| Spirochaetes | X | X |
| TM6 | X | X |
| TM7 | X | X |
| Verrucomicrobia | X | X |
| WS3 |  | X |
| ZB3 | X | X |
